# Two Decades of Gender Differences in Pornography Research Topics

**DOI:** 10.1007/s10508-025-03175-6

**Published:** 2025-06-19

**Authors:** Jingyuan Yu, Aliya Andrich, Max Schindler

**Affiliations:** Department of Economic Sciences and Media, Technische Universität Ilmenau, Ehrenbergstraße 29, 98693 Ilmenau, Germany

**Keywords:** Gender differences, Pornography, Bibliometrics, Thematic mapping, Longitudinal analysis

## Abstract

This exploratory study investigates the potential gender differences in research priorities, driven by factors such as established sexual stigma and traditional gender norms surrounding pornography. Using temporal and thematic analyses, we identify and examine research topics of 6,145 pornography studies published between 2001 and 2024. Our findings suggest that the lead author's gender may influence topic selection, with women exploring topics that have historically been underrepresented or overlooked. Furthermore, female-led publications have engaged with internet and web-related research earlier and more prominently. Additionally, our analyses outline the evolution of male- and female-led publication patterns in the field, highlighting a trend toward more balanced representation. This study aims to serve as a foundational basis for future research, encouraging more thorough investigations into gender disparities and their underlying causes within this domain. We also highlight the importance of longitudinal research to uncover potential gender disparities over time.

**Supplementary Information:**

The online version contains supplementary material available at 10.1007/s10508-025-03175-6.

Gender disparities in scientific research have been widely examined in (natural) science, technology, engineering, and mathematics (STEM; e.g., Taasoobshirazi & Carr, 2008), but less attention has been paid to smaller academic domains such as pornography studies. Moreover, existing bibliometric studies tend to focus on quantitative disparities between female and male researchers employing metrics such as research productivity and citation impact to assess their scientific performance (e.g., Goyanes et al., 2024; Griffin et al., 2018, 2023).^1^ Qualitative gender differences, including the selection of research topics, have also been documented, albeit to a much lesser extent (e.g., Lassiter et al., 2023), particularly within the context of pornography research. In this study, we conducted an exploratory analysis to provide an initial overview of the research agendas of female and male lead authors within the field of pornography research. Below, we outline the state of the art in the field and our motivation for anticipating gender disparities in research topics within this domain.

Studies have consistently shown that female and male scholars differ in their research topics. A large-scale analysis of 26 academic fields found that female researchers focus more on education, social inequality, and sexual violence, often using qualitative methods (Thelwall et al., 2019). A similar gender divide in topics appears in computational linguistics (Vogel & Jurafsky, 2012), library science (Bisaria & Jaiswal, 2019; Zhang et al., 2023), and management science (Nielsen et al., 2017). In biochemistry, genetics, and molecular biology, women tend to emphasize family-related terms, whereas men focus on methodologies, though no gender differences appear in physics (Pan & Kalinaki, 2015). These patterns align with the “people vs. things” explanation, where women gravitate toward social topics and men toward technical ones (Thelwall et al., 2019). Since topic selection influences funding outcomes (Hoppe et al., 2019), gender balance is crucial for equitable knowledge production. Additionally, including women as authors increases the likelihood of gender being considered in analyses (Nielsen et al., 2017), suggesting that diverse perspectives can promote broader social equity.

To our knowledge, there is little to no research on gender differences in the choice of topics within the field of pornography research. Pornography, explicit audiovisual content meant to arouse viewers (Hald, 2006; Wright, 2020), directly depicts female and male roles, offering script and behavioral models for learning about sexuality and gender relations (Bandura, 1969; Simon & Gagnon, 1986). Given that most of the commercial mainstream pornography portrays heterosexual encounters (Doring & Miller, 2022), these scripts often reflect gender stereotypes (Bridges et al., 2010) linking men to masculinity (e.g., assertiveness, sexual adventurism) and women to femininity (e.g., submissiveness, sexual modesty; Eagly, 1987; Siegel & Meunier, 2019). The asymmetrical gender roles present in pornography may influence how female and male scholars perceive and prioritize issues in their research, for instance, leading female authors to focus on representation and objectification.

Alongside traditional gender roles, gender differences in consumption of and attitudes toward pornographic content itself may influence the scientific output of pornography researchers. Some disparities are well-documented at the population level (Speed et al., 2021; Wright & Vangeel, 2019): men are exposed to sexually explicit materials earlier (Ševčíková et al., 2013) and consume pornography more frequently than women (Petersen & Hyde, 2010), while women tend to hold more negative views (Lykke & Cohen, 2015) and express greater ethical concerns about its social impact (Böhm et al., 2015). Additionally, individuals perceive gendered differences in pornography use that align with societal expectations (McElroy et al., 2023), reinforcing the idea that pornography consumption is a normative aspect of male, but not female, sexuality. In many cultures, female sexual desire and pornography consumption are subject to stigmatization, with pornography use among women often being perceived as deviant, inappropriate, and shameful (Marques, 2019). These normative gendered boundaries surrounding pornography use may affect female participation in pornography research and influence their choice of topics, as some may feel uncomfortable engaging with subjects considered taboo for women. Alternatively, female scholars may be more aware of this sexual stigma, leading to unique lines of inquiry that address shame, secrecy, or empowerment in different ways than men (Irvine, 2015).

Furthermore, the research agendas of female and male pornography scholars may be influenced by the historical context and social debates surrounding pornography at the time of their work. In the 1980s, some US feminists and researchers argued that pornography harmed women by perpetuating violence and power imbalances (e.g., MacKinnon & Dworkin, 1997). By the 1990s, third-wave feminists reframed pornography as a source of self-expression and empowerment (McElroy, 1995). Over the last 25 years, the rise of the internet, smartphones, and social media has further expanded pornography’s accessibility, resulting in more accepting attitudes among both men and women (Lykke & Cohen, 2015). In this shifting landscape, female and male scholars may approach pornography differently Female researchers may focus on the harm and sexual degradation of women or, alternatively, on sexual liberation and education, depending on the decade in which a study was conducted. Despite these historical debates and changing social norms, little to no longitudinal research has examined how the research agendas of female and male pornography scholars have diverged or converged over time.

Considering the above-mentioned arguments for potential gender differences, we conducted an exploratory bibliometric thematic investigation of the research topics examined by female and male pornography scholars using co-word analysis. In addition, this exploratory work provides a preliminary examination of temporal changes in the research agendas of female and male academics within this field over the past 24 years. The objective of this study is twofold: first, to establish a foundation for future research, and second, to encourage the development of more comprehensive studies that analyze gender differences and their causes in this field.

## Method

The data for our analyses were retrieved from the core database of Web of Science by searching keywords “pornography,” “pornographic,” and “sexually explicit material(s)” in the article title, keywords, or abstract. This resulted in a dataset of 6145 journal articles and conference papers published between 2001 and 2024. The gender of the first authors was identified by a combination of automatic detection (by software Genderize.io) and manual annotation (by the authors of this paper), following an inter-rater reliability check on a random subset of the data (Krippendorff’s alpha of 0.94). The dataset contained 2912 articles (47%) that were first-authored by women and 3233 articles (53%) that were first-authored by men. More details regarding gender identification are available on the supplementary information.

To explore potential gender differences in research topics, the collected data was divided into two subsets based on the gender (i.e., male and female) of the first author. We followed the methodology proposed by Cobo et al. (2011) to identify research topics within each subset. First, a co-word network was constructed based on the co-occurrence of keywords. Next, a clustering algorithm was used to detect the clusters of the network. Specifically, we employed the walktrap algorithm, which uses random walks to measure the distance between vertices (i.e., keywords) and has been shown to be effective and accurate (Pons & Latapy, 2006). Each of the detected clusters is understood as a research theme (Cobo et al., 2011).

**Fig. 1:**
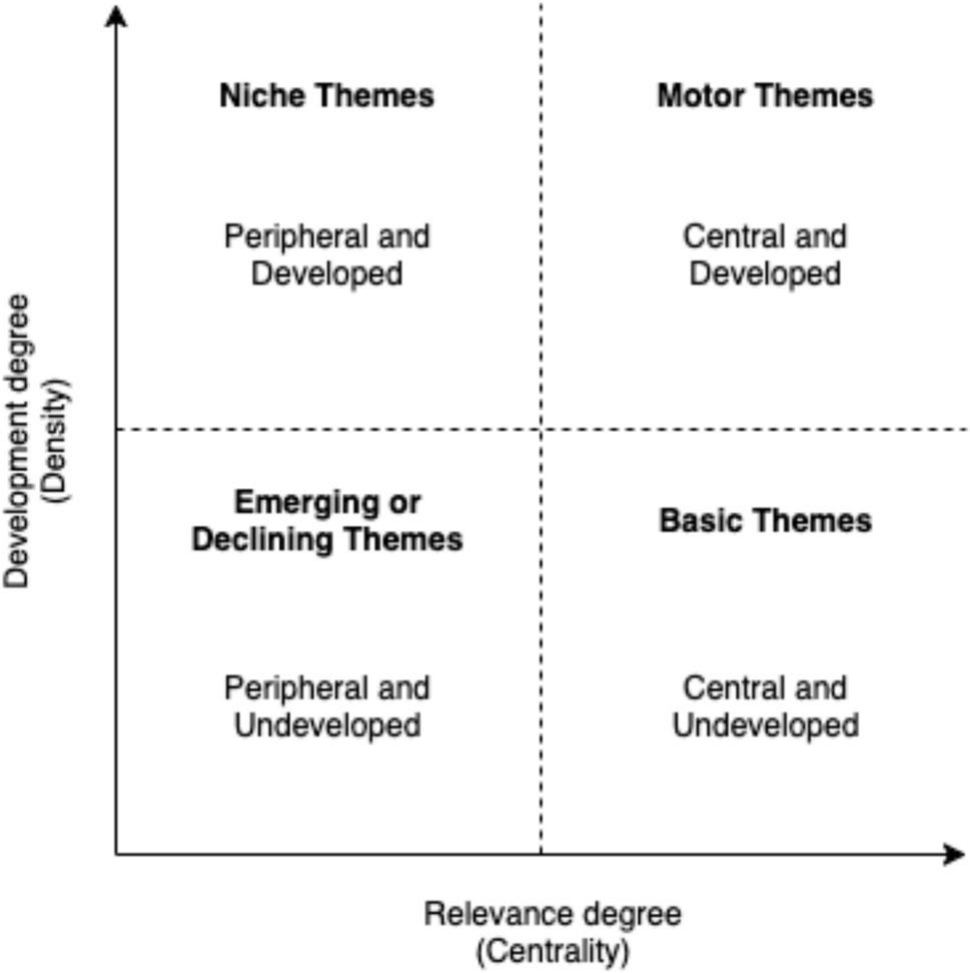
Conceptual thematic map of centrality and density

Based on these clusters, two metrics were calculated: Callon’s centrality and density (Callon et al., 1991). Centrality quantifies the extent of a cluster’s connections with other clusters, with higher values indicating a greater role in linking different research topics. A topic with high centrality is considered influential in shaping the broader research field. Therefore, centrality can measure the relevance and importance of a topic in the field (Cobo et al., 2011). Density measures the strength of internal connections among the keywords within a cluster. A higher density suggests that the research topic is internally coherent and integrated. Therefore, density can be understood as a measure of the topic’s development itself (Cobo et al., 2011). According to these two metrics, a two-dimensional thematic map can be visualized (see Fig. 1). In this conceptual map, the *x*-axis measures a theme’s relevance to other research areas (centrality), and the *y*-axis measures how cohesively that theme is organized (density; Giannakos et al., 2020; Yu & Muñoz-Justicia, 2020). The upper right quadrant contains “motor themes” that are both central to the field and internally coherent; the upper left quadrant captures specialized but less-connected areas (Giannakos et al., 2020; Yu & Muñoz-Justicia, 2020). Themes in the lower left quadrant are neither highly connected nor well-developed and may be emerging or declining; the lower right quadrant highlights underdeveloped but broadly relevant themes that could eventually shape the direction of the entire research field (Giannakos et al., 2020; Yu & Muñoz-Justicia, 2020). The thematic map was constructed in R using the *bibliometrix* package (Aria & Cuccurullo, 2017).

## Results

In this section, we first follow a common approach in bibliometric studies (e.g., Trillo-Domínguez & De-Moya-Anegón, 2022; Yu & Muñoz-Justicia, 2020), identifying the most prolific authors by publication count, spotlighting those who have notably shaped the field of pornography studies. Next, we review the top journals in which female and male authors published the most, emphasizing differences and similarities in their choice of outlets. We also examine the evolution of the proportion of articles with women as first authors over time. Subsequently, we present our main analysis of the research topics of female and male first authors. Finally, we provide a corresponding discussion of our findings and future research directions.

In our dataset, the top three most prolific male first authors were Paul Wright (72 first author articles), Samuel Perry (30 first author articles), and Joshua Grubbs (23 first author articles). The top three most prolific female first authors were Beata Bothe (20 first author articles), Carmen Cusack (17 first author articles), and Ethel Quayle (12 first author articles).

Table 1 shows the top 20 journals that have published the most articles by gender. Overall, many key journals (e.g., *Archives of Sexual Behavior, Journal of Sex Research*) appeared in both the male and female first-author datasets, indicating important common outlets within pornography research. However, certain journals were found exclusively in the male set (e.g., *PLoS ONE, Journal of Homosexuality, Frontiers in Psychology*), while others appeared only in the female dataset (e.g., *Sex Education, Violence Against Women, Feminist Media Studies*). These exclusive journal preferences may suggest that female authors may be emphasizing issues like feminist discourse around pornography, whereas male authors may be publishing more on broader, interdisciplinary topics.

**Table 1:** The top 20 journals according to the number of articles with female and male first authors

Male		Female	
Journal	Articles	Journal	Articles
Archives of Sexual Behavior	141 (4.36%)	Archives of Sexual Behavior	96 (3.30%)
Journal of Sex Research	64 (1.98%)	Journal of Sex Research	67 (2.30%)
Sexual Health & Compulsivity	61 (1.89%)	Sexuality and Culture	52 (1.79%)
Sexuality & Culture	49 (1.52%)	Sexualities	44 (1.51%)
Journal of Behavioral Addictions	45 (1.39%)	Sexual Health & Compulsivity	38 (1.30%)
Sexualities	42 (1.30%)	Journal of Behavioral Addictions	34 (1.17%)
Journal of Sex & Marital Therapy	35 (1.08%)	Journal of Sexual Medicine	32 (1.10%)
Sexual Abuse	30 (0.93%)	Journal of Interpersonal Violence	27 (0.93%)
Computers in Human Behavior	27 (0.84%)	Journal of Sexual Aggression	26 (0.89%)
Journal of Homosexuality	27 (0.84%)	Journal of Sex & Marital Therapy	25 (0.86%)
Journal of Sexual Medicine	26 (0.80%)	Sex Education	25 (0.86%)
International Journal of Sexual Health	22 (0.68%)	International Journal of Environmental Research and Public Health	24 (0.82%)
PLoS One	20 (0.62%)	Computers in Human Behavior	23 (0.79%)
Addictive Behaviors	19 (0.59%)	Sexuality Research and Social Policy	23 (0.79%)
Sexuality Research and Social Policy	19 (0.59%)	Sexual Abuse	22 (0.76%)
Frontiers in Psychology	16 (0.49%)	Violence Against Women	22 (0.76%)
International Journal of Mental Health and Addiction	15 (0.46%)	Current Addiction Reports	21 (0.72%)
International Journal of Environmental Research and Public Health	14 (0.43%)	Feminist Media Studies	20 (0.69%)
Journal of Interpersonal Violence	14 (0.43%)	Sexual Health	18 (0.62%)
Journal of Sexual Aggression	14 (0.43%)	Journal of Child Sexual Abuse	17 (0.58%)
Total Number of Articles	3233	Total Number of Articles	2912

The relative number of articles by gender is provided in parentheses

**Fig. 2:**
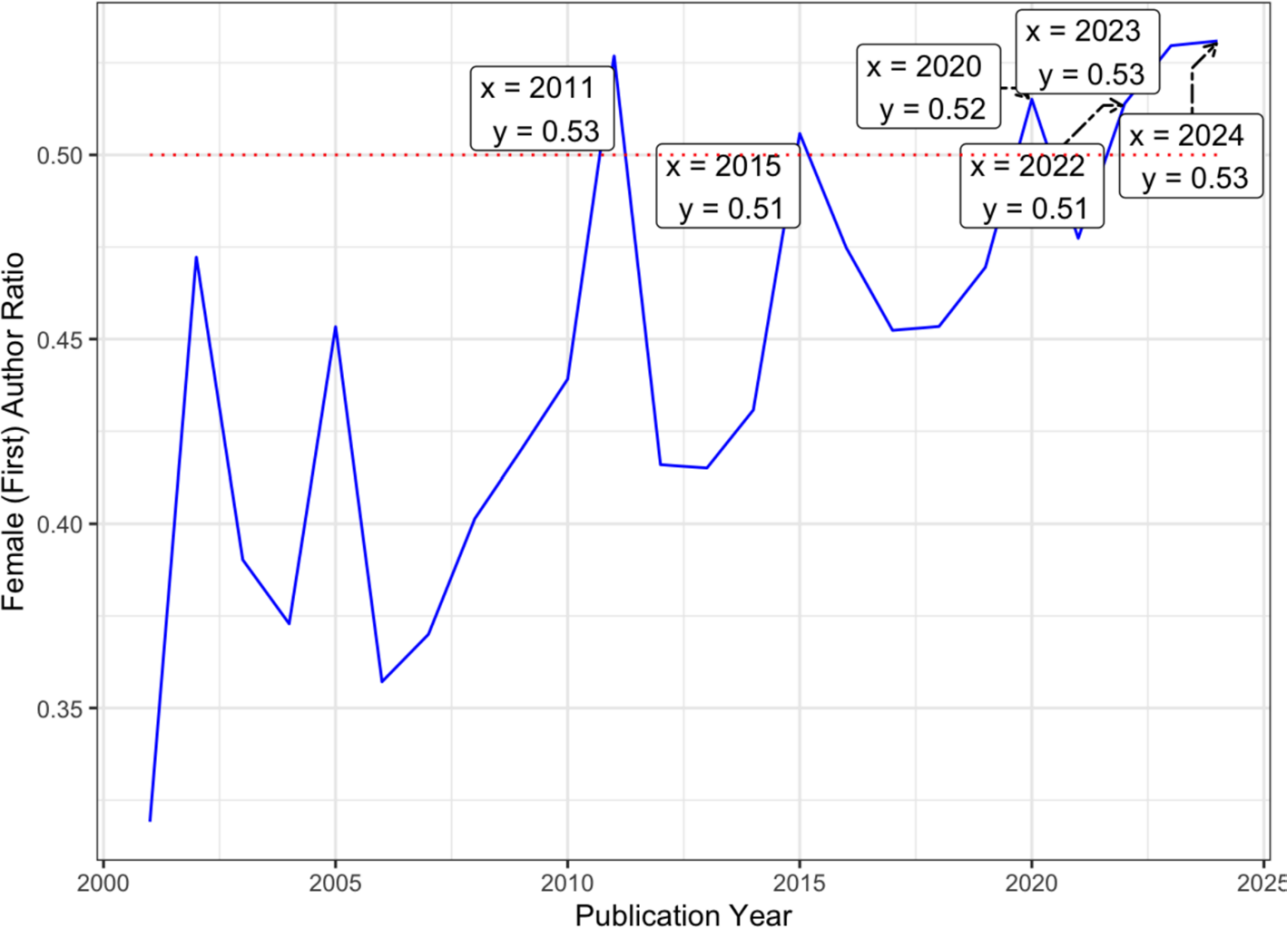
The percentage of articles with women as first authors by year. *Note.* The dashed line indicates the point at which 50% of publications have female first authors

We calculated the yearly ratios of female first authors, which reflects the proportion of articles with women as first authors (Fig. 2). While women led fewer than half of the publications in the early 2000s, from the beginning of the next decade, this proportion rose steadily. Despite the presence of certain fluctuations, the proportion of articles with female first authors stabilized slightly above 50% after 2020. This shift may reflect a positive progress toward gender balance in pornography research, with recent years consistently showing female first authors at or above parity with men, although the COVID-19 pandemic might have influenced research priorities across disciplines (Nasir et al., 2020; Pourhatami et al., 2021).

**Fig. 3:**
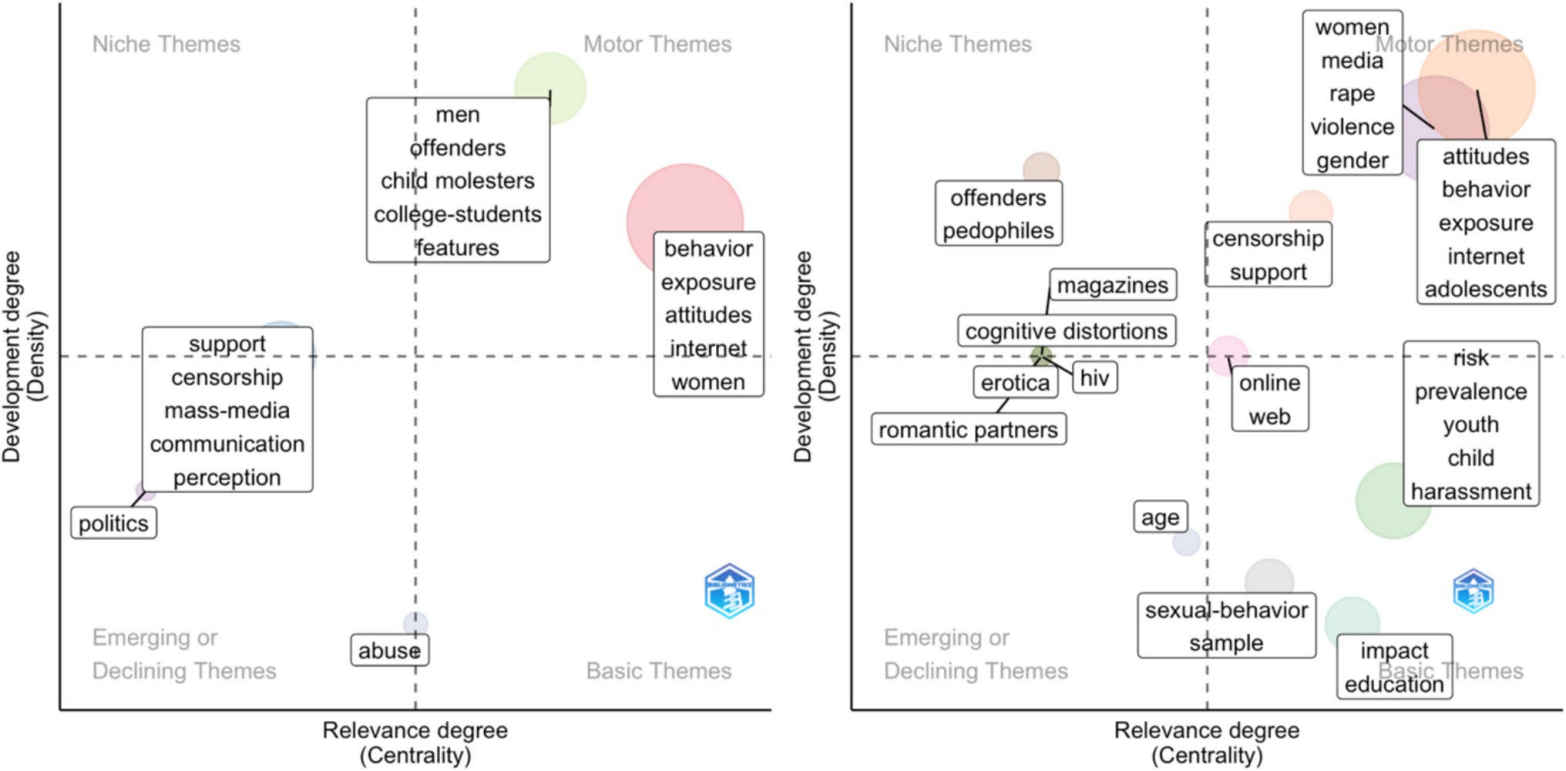
Thematic map for male (left) and female (right) first author articles published from 2001 to 2010. *Note.* Each circle represents a single research theme

These distinct periods also established the foundation for our main analysis of over-time differences, in which we divided the data into three time periods based on the proportion of female first authors. For the main analysis of gender disparities in pornography, scholars’ research topics were identified through a co-word analysis of Web of Science’s Keyword Plus.^2^ During the initial period from 2001 to 2010, when male researchers dominated the field (see Fig. 3), female first-authored papers appeared to have a much more diverse set of research interests than papers with male first authors. Nevertheless, they shared several topics within this period. Specifically, studies on exposure to and attitudes toward pornography were among the motor themes (i.e., central and developed) for both women and men; however, the former focused on adolescents (attitudes, behavior, exposure, internet, adolescents) and the latter on women (behavior, exposure, attitudes, internet, women). Censorship was the central and developed topic for female authors (censorship, support), but a rather niche topic (i.e., developed but peripheral) for their male counterparts, who placed greater emphasis on perception and the role of mass media and communication (support, censorship, mass media, communication, perception). In contrast, research on offenders was a central topic (i.e., motor theme; men, offenders, child molesters, college students, features) in male-led publications, while a similar topic in female-led publications (offenders, pedophiles) remained relevant but isolated (i.e., niche topic). During this time, male-led research had two emerging or declining research foci in abuse and politics that were peripheral and undeveloped. Female-led research during this period was more granular, including research on romantic partners, erotica, HIV, magazines, cognitive distortions, and age as emerging or declining themes, and education and its impact (impact, education), sexual behavior (sexual behavior, sample), the online aspect of pornography (online, web), and its risks associated with children (risk, prevalence, youth, child, harassment) as basic research themes.

**Fig. 4:**
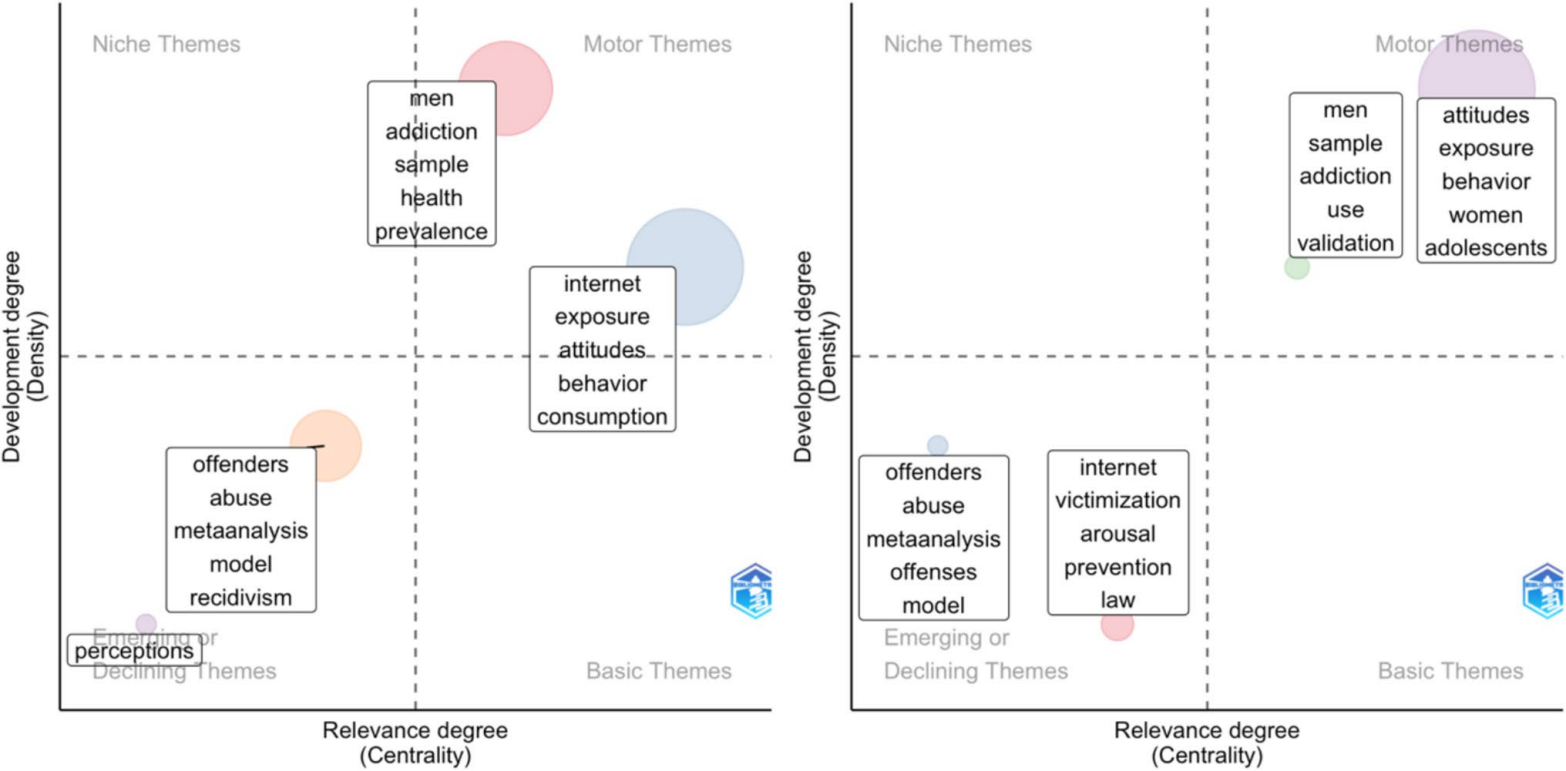
Thematic map for male (left) and female (right) first author articles published from 2011 to 2019. *Note.* Each circle represents a single research theme

The second period, from 2011 to 2019, in which female representation increased but remained relatively low, reflected a phase of consolidation and refinement, with research becoming more structured within clearly defined fields (see Fig. 4). A common focus included research on attitudes toward pornography, which represented a developed and central research interest (i.e., motor theme). In male-led publications, this theme was often explored alongside research on the internet and general consumption patterns (internet, exposure, attitudes, behavior, consumption), while female-led publications tended to examine it in relation to women and adolescents (attitudes, exposure, behavior, women, adolescents). Another key shared research area during this period was addiction, particularly with a focus on men. Being central and developed motor themes, these topics formed the core of pornography research at the time for both women (men, sample, addiction, use, validation) and men (men, addiction, sample, health, prevalence). In contrast, studies on offenders and abuse declined compared to the first period, with both male- (offenders, abuse, meta-analysis, model, recidivism) and female-led (offenders, abuse, meta-analysis, offenses, model) publications becoming less cohesive, indicating waning interest or a shift in focus. Additionally, a distinct area of interest in male-led publications was research topic perceptions, though this field remained undeveloped and peripheral (i.e., emerging or declining theme). In female-led publications, emerging but still nascent research areas included pornography regulation and victimization (internet, victimization, arousal, prevention, law).

**Fig. 5:**
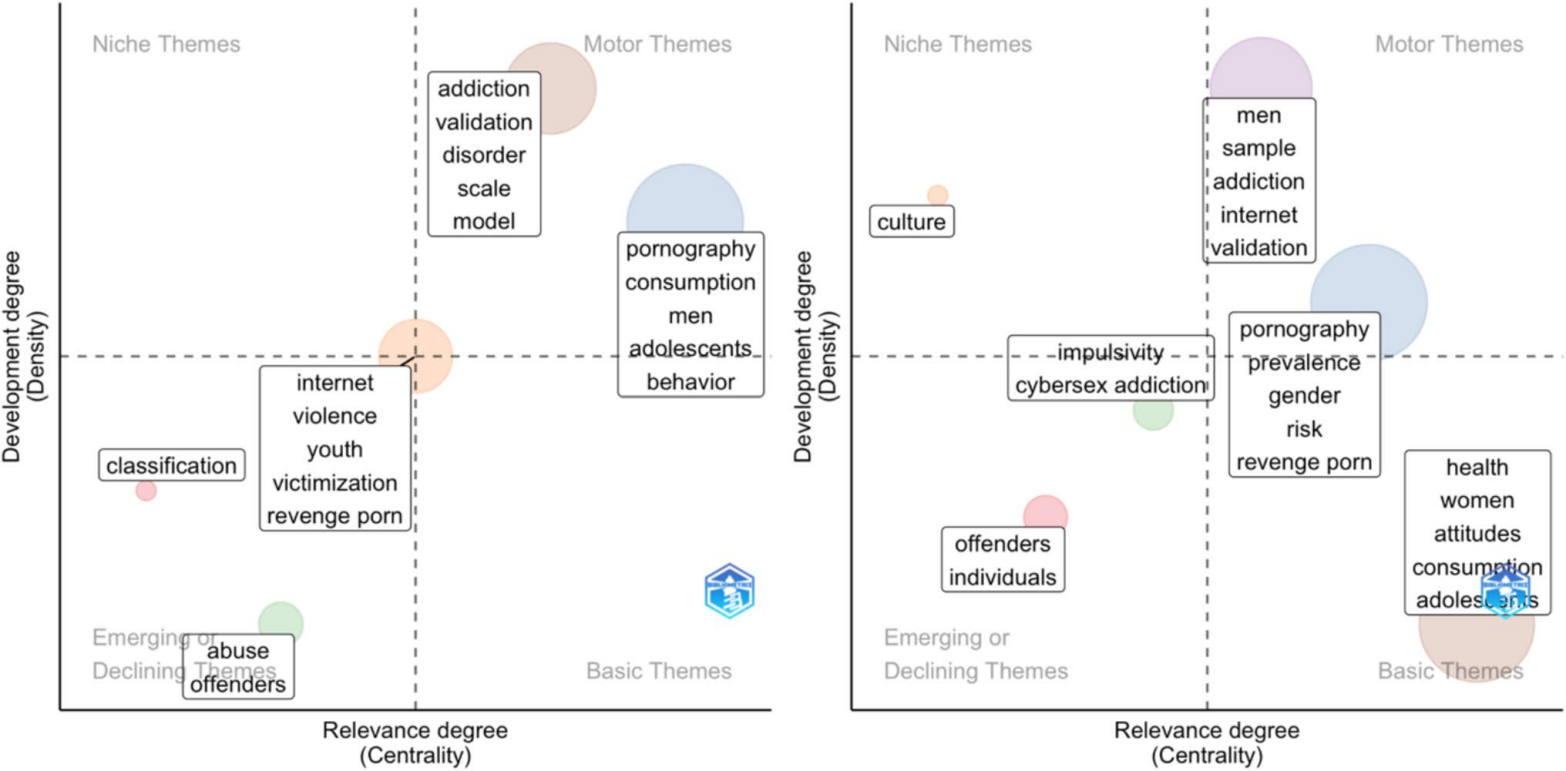
Thematic map for male (left) and female (right) first author articles published from 2020 to 2024. *Note.* Each circle represents a single research theme

In the final period of this study, from 2020 to 2024, during which female researchers achieved a more stable presence, (see Fig. 5), research on offenders and abuse continued to decline in relevance in both male- (abuse, offenders) and female-led (offenders, individuals) publications. As in previous periods, addiction remained one of the motor themes for men (addiction, validation, disorder, scale, model) as well as women (men, sample, addiction, internet, validation). Pornography consumption had become an established motor theme in articles with male first authors (pornography, consumption, men, adolescents, behavior), whereas in articles with female first authors, the topic was central but undeveloped (i.e., basic theme; health, women, attitudes, consumption, adolescents). Risk factors associated with pornography had become a relevant and somewhat developed theme (i.e., motor theme) in female-led publications (pornography, prevalence, gender, risk, revenge porn). Conversely, a comparable topic in publications led by men (internet, violence, youth, victimization, revenge porn) could be characterized as gaining traction and demonstrating potential as a future motor theme.

For female-led research, a distinct yet less pronounced niche theme emerged focused on cultural aspects of pornography (culture). In contrast, male-led research demonstrated a growing interest in more technical approaches (classification).

## Discussion

In this commentary, we systematically analyze qualitative differences in the research agendas of female and male pornography scholars, examining how these patterns evolved over a twenty-four-year period. Our findings suggest that the gender of the lead author may affect topic selection, consistent with trends identified in other academic disciplines (e.g., Bisaria & Jaiswal, 2019; Lassiter et al., 2023; Nielsen et al., 2017; Thelwall et al., 2019; Vogel & Jurafsky, 2012; Zhang et al., 2023). However, our research design, which involved both temporal and thematic analyses, allows us to identify certain nuances that extend beyond this general pattern. More specifically, while we find that female and male first authors often investigate overlapping research topics, clear differences emerge when analyzing changes in research agendas over time. These gender differences become particularly pronounced when considering topic coherence and their broader relevance to the field. In particular, over the three time periods examined here (2001–2010, 2011–2019, and 2020–2024), one prominent finding is that women’s research agendas tend to be more diverse and somewhat more socially oriented. During the 2000s, female-led studies emphasized violence, harassment, rape, censorship, and peripheral issues (e.g., romantic partners, HIV, age factors), whereas male-led studies centrally addressed offenders and emphasized communication and perception. In the 2010s, attitudes and addiction emerged as common themes for both genders; however, women focused more on women and adolescents, while men examined internet use and general consumption patterns. In the most recent period, addiction remained a core theme for both, though female-led studies increasingly explored risk factors, gender dynamics, and cultural aspects of pornography. These patterns suggest that while there is some thematic convergence, such as a shared emphasis on attitudes, addiction and consumption, clear differences exist. These findings also support previous research showing that women tend to explore topics that are historically underrepresented or overlooked (Roy et al., 2023). Conversely, we find little support for the notion that female authors predominantly focus on non-technical themes (Haines et al., 2020). Instead, our results suggest that female-led publications have engaged with internet and web-related research earlier and more prominently.

In addition to our main findings, we discover quantitative gender differences in pornography research, highlighting a potential trend toward more balanced representation. This mirrors similar patterns in other research fields, where women have historically been underrepresented as lead authors, with the gender gap gradually closing (Joanis & Patil, 2022; Zettler et al., 2017; Zhao et al., 2023). Furthermore, the gradual rise in female-authored work also appears to broaden the field’s scope, supporting suggestions that a more diverse authorship may introduce new perspectives and lines of inquiry (Nielsen et al., 2017).

Our results echo broader findings on how gender norms and research interests intersect in pornography studies. For example, female-led publications’ emphasis on harm, risk, and issues affecting women and adolescents may reflect the heightened stigma often associated with women’s engagement in sexual content (Marques, 2019). Meanwhile, men’s consistent focus on offenders and broader consumption patterns may reflect differing cultural scripts, such as the notion that pornography consumption is predominantly a male activity (McElroy et al., 2023). Over time, shifts in feminist views regarding pornography, from considering it harmful (MacKinnon & Dworkin, 1997) to potentially empowering (McElroy, 1995), may have further shaped how female scholars approach and interpret pornography research.

The present study offers only a preliminary perspective on this issue. Future research should first confirm our findings with larger samples and rigorous statistical methods. If these differences persist, scholars could then explore their underlying causes, whether driven by sexual stigma, gender norms, or broader social contexts. Although large-scale analyses such as this study are important, cross-cultural comparisons, along with qualitative inquiries (e.g., interviews with researchers), could provide a more detailed view on how and why these differences arise. One of the major limitations of this study, our reliance on a binary view of researcher gender, overlooks the complexity of gender identities. Future research should consider contacting scholars to inquire about their gender identities (e.g., as done by Lassiter et al., 2023) in order to draw more inclusive conclusions. Additionally, factors such as culture, race, and religion (Patterson & Price, 2012) may influence researchers’ agendas but are difficult to study at scale without collecting sensitive personal data, posing significant ethical and practical challenges. Last but not least, our research focused exclusively on the gender of first authors, as they are generally considered to be the person who has contributed the most to the paper (Joanis & Patil, 2022; Shang et al., 2022). The gender of co-authors was not considered within our research scope, despite their potential influence in shaping research directions (e.g., supervisors, funding recipients). Future studies should expand our scope onto a more nuanced analysis of inter-gender collaboration and, if possible, clarify the roles that drive scientific production.

## Supplementary Information

Below is the link to the electronic supplementary material.

Supplementary file1 (PDF 632 kb)

## Data Availability

Research data for this study are accessible via Web of Science; research materials (e.g., reproducible report) are available in supplementary information.

## Footnotes

1 In the context of this study, gender is viewed as a binary concept. Admittedly, this is a flawed representation of the concept, as it overlooks the complexity and diversity of gender identities beyond traditional categories. It also runs the risk of marginalizing non-binary, genderqueer, and transgender individuals, potentially limiting the inclusivity of research findings. We discuss this limitation in the context of our findings.

2 https://support.clarivate.com/ScientificandAcademicResearch/s/article/KeyWords-Plus-generation-creation-and-changes?language=en_US

## Code Availability

Code used in this study is available in supplementary information.
