# Supplementary material for: Two Decades of Gender Differences in Pornography Research Topics
Source: Arch Sex Behav. 2025 Jun 19;54(6):1995–2003. doi: 10.1007/s10508-025-03175-6 (PMC12283780; doi:10.1007/s10508-025-03175-6)
Supplement: Supplementary file 1 — Supplementary file1 (PDF 632 kb) [file 10508_2025_3175_MOESM1_ESM.pdf]

Supplementary Material

Two Decades of Gender Differences in Pornography Research Topics

Anonymous Author for Peer Review

Anonymous Institution for Peer Review

Data Collection and Gender Identification

The data for our analysis was obtained from the core database of Web of Science (WOS) by searching keywords "pornography", "pornographic", and "sexually explicit material(s)". Two rounds of data collection were conducted along with the development of our study. The first round took place in March 2021, in this round we collected all the WOS core database indexed articles between 2001 and 2021 (March). The second round took place in November 2024, here we collected all the WOS core database indexed articles from 2021 to 2024 (November).

In the first round, a total of 4,097 articles were collected, and in the second round, 2,160 articles were gathered. To merge the data from both rounds and account for duplicated items (between January and March 2021, since WOS does not offer filters for exact dates), we removed the 2021 (publication year) data from the first round dataset. We then combined the two datasets, resulting in a total of 6,145 unique items.

The identification of first-author's gender was conducted in two rounds, respectively. In the first round, software program "genderize.io" was used to detect the gender of the first author. The software also provided the accuracy of its identification, a name was assigned though manual coding if the respective accuracy was below 90%. Two coders assessed the name manually, and across fifty manually coded names, inter-coder reliability was 1. In this round, among the collected 4,097 items, 2,985 were identified by software, and 1,112 were coded manually.

After completing the second round data collection, the collected data was distributed to three coders for manual coding. The inter-rater reliability (Krippendorff's alpha) was 0.94. Upon merging the coded data, our final dataset consisted of 2,912 articles with a female first author and 3,233 articles with a male first author.

Reproducible Report

Mapping Gender Differences in Pornography Research: A Bibliometric Analysis

Anonymous Author

Apr 2025

Load data

```
library(tidyverse)
library(bibliometrix)

load("data_preparation/final_data.RData")
```

Subset the data into male/female set

```
male <- data %>% filter(gender == "male")
female <- data %>% filter(gender == "female")
```

```
cat("total number of documents:", nrow(data))
```

```
## total number of documents: 6145
```

```
cat("total number of male documents:", nrow(male))
```

```
## total number of male documents: 3233
```

```
cat("total number of female documents:", nrow(female))
```

```
## total number of female documents: 2912
```

Top 3 authors

ranked by number of first author articles, as we wrote in the original manuscript.

Male

```
male_top_author <- male %>%
  mutate(first_author = gsub(";.*", "", AU)) %>% #remove everything after semicolon, and extract only t
  count(first_author) %>%
  arrange(-n)

male_top_author %>% head(3)
```

```
##   first_author  n
## 1    WRIGHT PJ 72
## 2     PERRY SL 30
## 3    GRUBBS JB 23
```

Female

```
female_top_author <- female %>%
  mutate(first_author = gsub(";.*", "", AU)) %>%
  count(first_author) %>%
  arrange(-n)

female_top_author %>% head(3)
```

```
##   first_author  n
## 1      BOTHE B 20
## 2    CUSACK CM 17
## 3     QUAYLE E 12
```

Top 20 journals

Male

```
male_des_results <- male %>%
  mutate(DB = "wos") %>%
  biblioAnalysis(sep = ";")

male_des_results$Sources[1:20]
```

```
## SO
##                                          ARCHIVES OF SEXUAL BEHAVIOR
##                                                                  141
##                                              JOURNAL OF SEX RESEARCH
##                                                                   64
##                                     SEXUAL HEALTH \\& COMPULSIVITY
##                                                                   61
##                                             SEXUALITY AND CULTURE
##                                                                   49
##                                   JOURNAL OF BEHAVIORAL ADDICTIONS
##                                                                   45
##                                                        SEXUALITIES
##                                                                   42
##                                   JOURNAL OF SEX \\& MARITAL THERAPY
##                                                                   35
##                    SEXUAL ABUSE-A JOURNAL OF RESEARCH AND TREATMENT
##                                                                   30
##                                        COMPUTERS IN HUMAN BEHAVIOR
##                                                                   27
##                                          JOURNAL OF HOMOSEXUALITY
##                                                                   27
##                                        JOURNAL OF SEXUAL MEDICINE
##                                                                   26
##                              INTERNATIONAL JOURNAL OF SEXUAL HEALTH
##                                                                   22
##                                                          PLOS ONE
##                                                                   20
##                                              ADDICTIVE BEHAVIORS
##                                                                   19
##                                SEXUALITY RESEARCH AND SOCIAL POLICY
```

```
##                                                                    19
##                                               FRONTIERS IN PSYCHOLOGY
##                                                                    16
##                INTERNATIONAL JOURNAL OF MENTAL HEALTH AND ADDICTION
##                                                                    15
## INTERNATIONAL JOURNAL OF ENVIRONMENTAL RESEARCH AND PUBLIC HEALTH
##                                                                    14
##                                         JOURNAL OF INTERPERSONAL VIOLENCE
##                                                                    14
##                                              JOURNAL OF SEXUAL AGGRESSION
##                                                                    14
```

Female

```
female_des_results <- female %>%
  mutate(DB = "wos") %>%
  biblioAnalysis(sep = ";")

female_des_results$Sources[1:20]
```

```
## SO
##                                              ARCHIVES OF SEXUAL BEHAVIOR
##                                                                    96
##                                                  JOURNAL OF SEX RESEARCH
##                                                                    67
##                                                   SEXUALITY AND CULTURE
##                                                                    52
##                                                            SEXUALITIES
##                                                                    44
##                                          SEXUAL HEALTH \\& COMPULSIVITY
##                                                                    38
##                                         JOURNAL OF BEHAVIORAL ADDICTIONS
##                                                                    34
##                                               JOURNAL OF SEXUAL MEDICINE
##                                                                    32
##                                         JOURNAL OF INTERPERSONAL VIOLENCE
##                                                                    27
##                                              JOURNAL OF SEXUAL AGGRESSION
##                                                                    26
##                                        JOURNAL OF SEX \\& MARITAL THERAPY
##                                                                    25
##                            SEX EDUCATION-SEXUALITY SOCIETY AND LEARNING
##                                                                    25
## INTERNATIONAL JOURNAL OF ENVIRONMENTAL RESEARCH AND PUBLIC HEALTH
##                                                                    24
##                                              COMPUTERS IN HUMAN BEHAVIOR
##                                                                    23
##                                      SEXUALITY RESEARCH AND SOCIAL POLICY
##                                                                    23
##                        SEXUAL ABUSE-A JOURNAL OF RESEARCH AND TREATMENT
##                                                                    22
##                                                   VIOLENCE AGAINST WOMEN
##                                                                    22
##                                                CURRENT ADDICTION REPORTS
```

```
##                                                    21
##                              FEMINIST MEDIA STUDIES
##                                                    20
##                                      SEXUAL HEALTH
##                                                    18
##                        JOURNAL OF CHILD SEXUAL ABUSE
##                                                    17
```

Female author rate over the years

three time periods:

p1 = 2001 - 2010

p2 = 2011 - 2019

p3 = 2020 - 2024

```
data %>%
  count(PY, gender) %>%
  group_by(PY) %>%
  mutate(sum = sum(n)) %>%
  ungroup() %>%
  mutate(rate = n/sum) %>%
  filter(gender == "female") %>%
  mutate(my_label = ifelse(rate >= 0.5,
                           paste("x =", PY, "\n","y =", round(rate, 2)),
                           "")) %>%
  ggplot(aes(x = PY, y = rate)) +
  geom_line(color = "blue") +
  ggrepel::geom_label_repel(aes(label = my_label),
                            box.padding = 0.2, max.overlaps = Inf,
                            segment.linetype = 6, segment.curvature = -1e-20,
                            arrow = arrow(length = unit(0.015, "npc"))) +
  geom_line(aes(y = 0.50), linetype = "dashed", col = "red") +
  ylab("Female (First) Author Ratio") +
  xlab("Publication Year") +
  theme_bw()
```

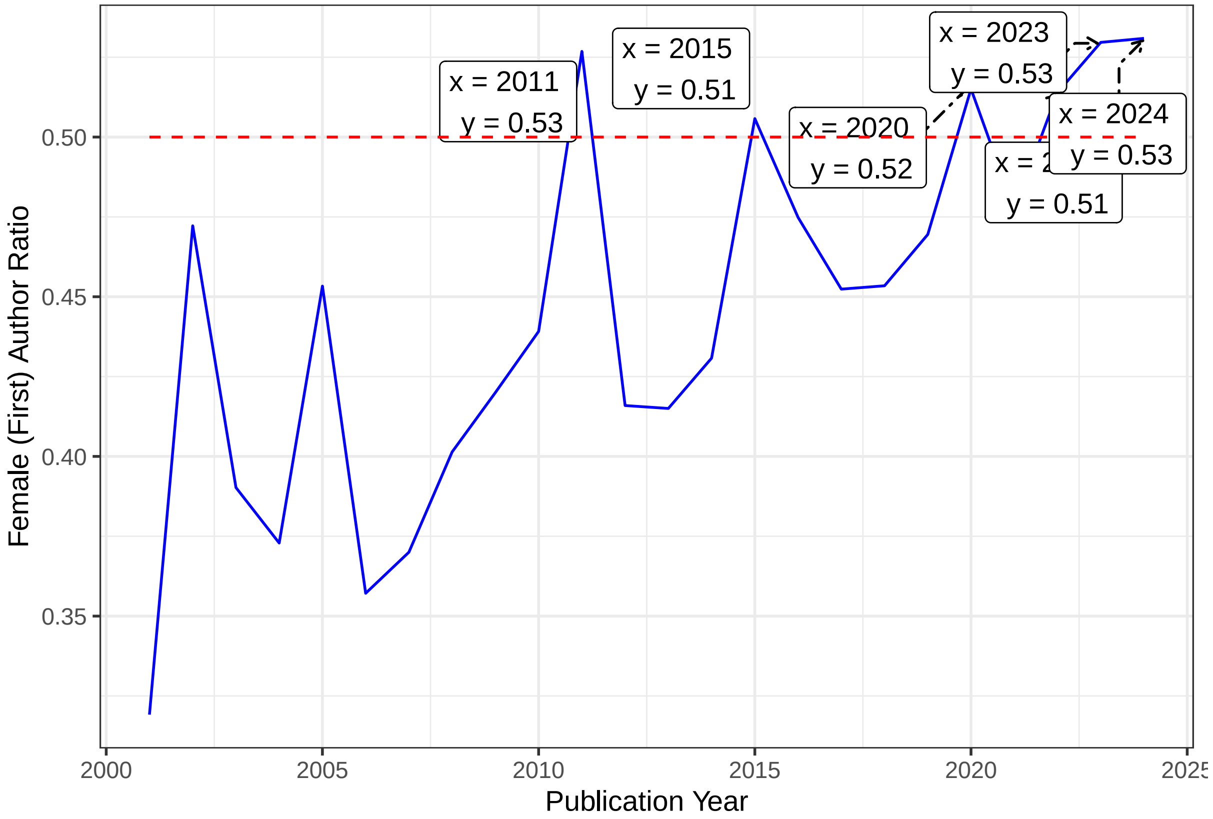

Split the male/female data into different subsets (=time periods)

```
male_p1 <- male %>% filter(PY <= 2010)
male_p2 <- male %>% filter(PY > 2010 & PY < 2020)
male_p3 <- male %>% filter(PY >= 2020)

female_p1 <- female %>% filter(PY <= 2010)
female_p2 <- female %>% filter(PY > 2010 & PY < 2020)
female_p3 <- female %>% filter(PY >= 2020)
```

Thematic map

Overall (2001-2024, NOT included in the paper, interpretation see next chapter)

Male

```
male_overall <- male %>%
  thematicMap(field = "ID", n=300, minfreq = 10, stemming = TRUE,
              size = 0.3, n.labels = 5)

plot(male_overall$map)
```

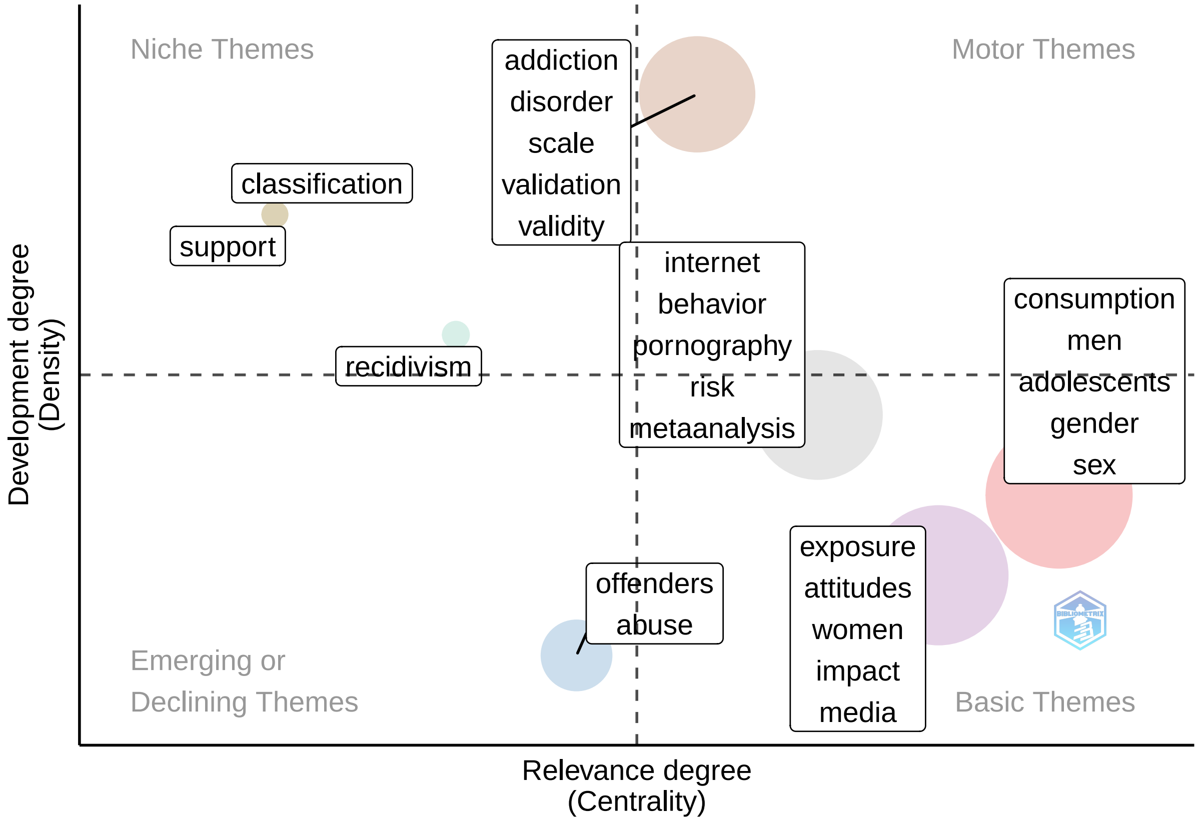

Female

```r
female_overall <- female %>%
  thematicMap(field = "ID", n=300, minfreq = 10, stemming = TRUE,
              size = 0.3, n.labels = 5)

plot(female_overall$map)
```

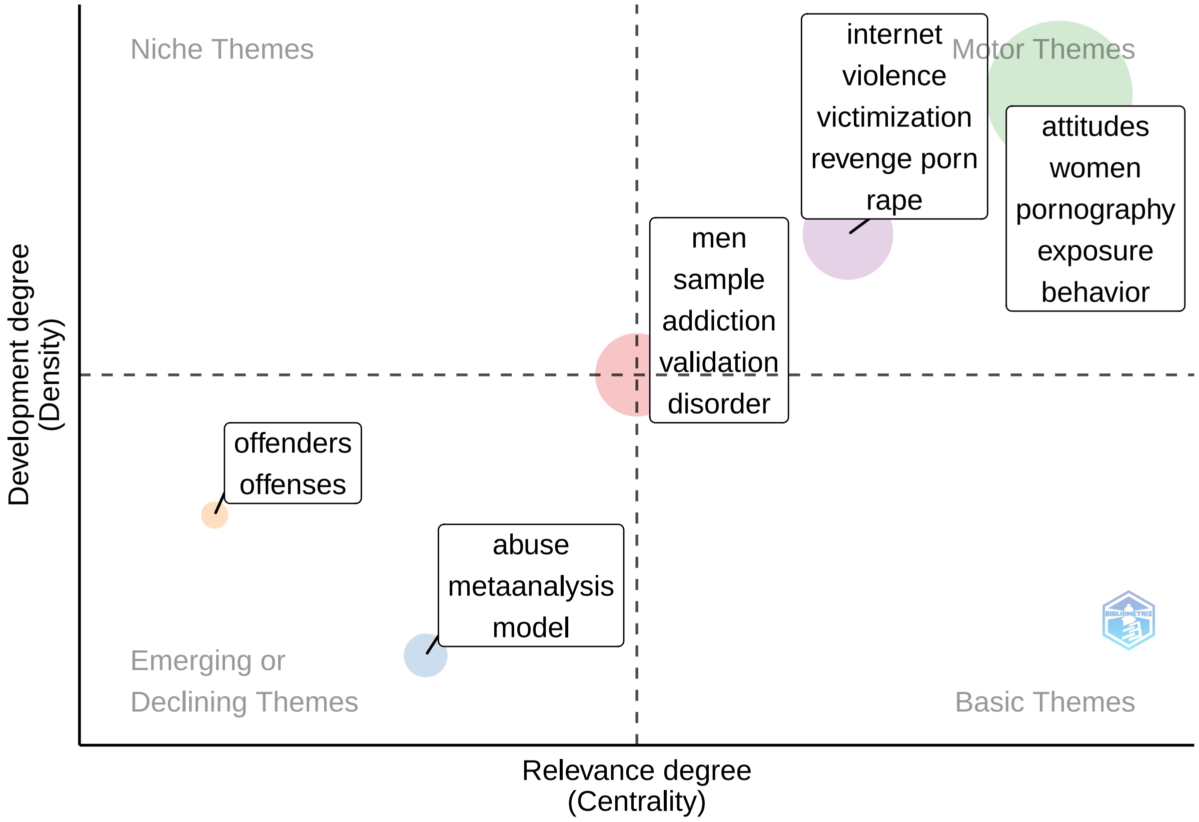

P1 (2001-2010)

Male

```r
# pmp1 = plot male period 1

pmp1 <- male_p1 %>%
  thematicMap(field = "ID", n=300, minfreq = 10, stemming = TRUE,
              size = 0.3, n.labels = 5)

plot(pmp1$map)
```

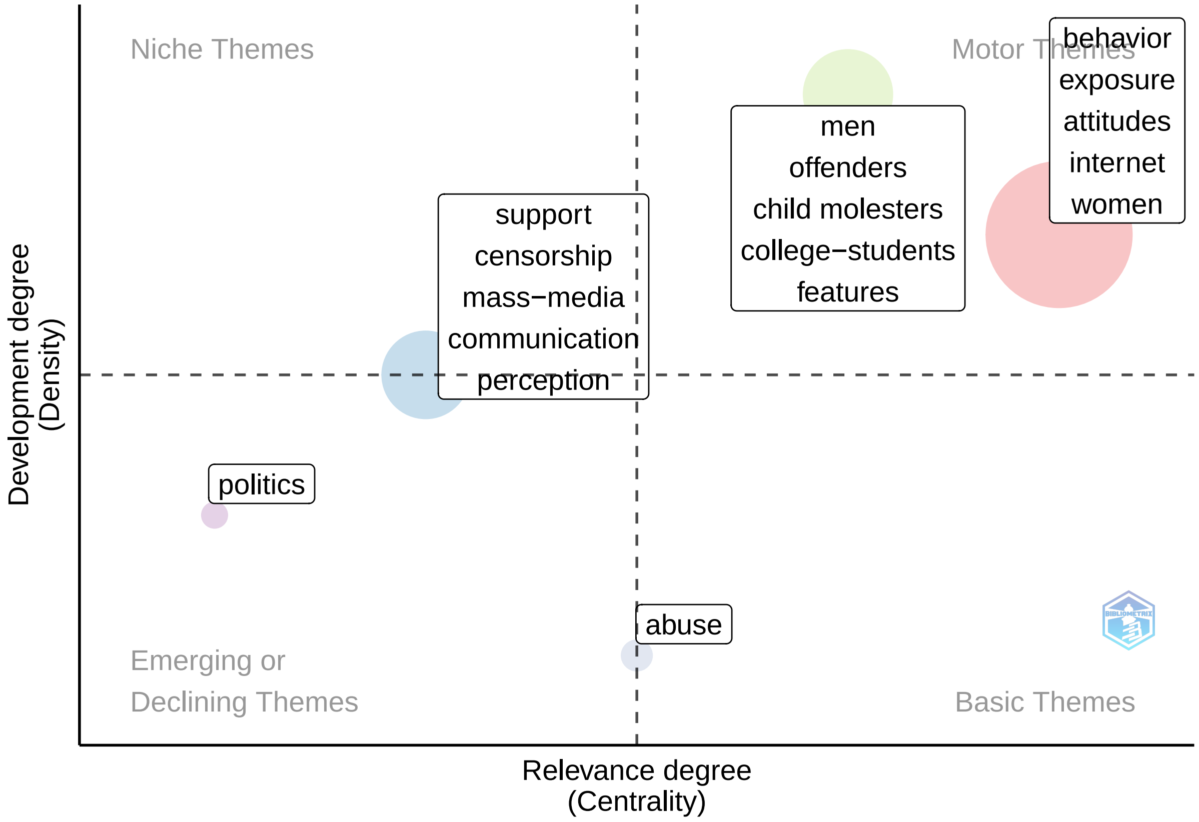

Female

```
# pfp1 = plot female period 1

pfp1 <- female_p1 %>%
  thematicMap(field = "ID", n=300, minfreq = 10, stemming = TRUE,
              size = 0.3, n.labels = 5)

plot(pfp1$map)
```

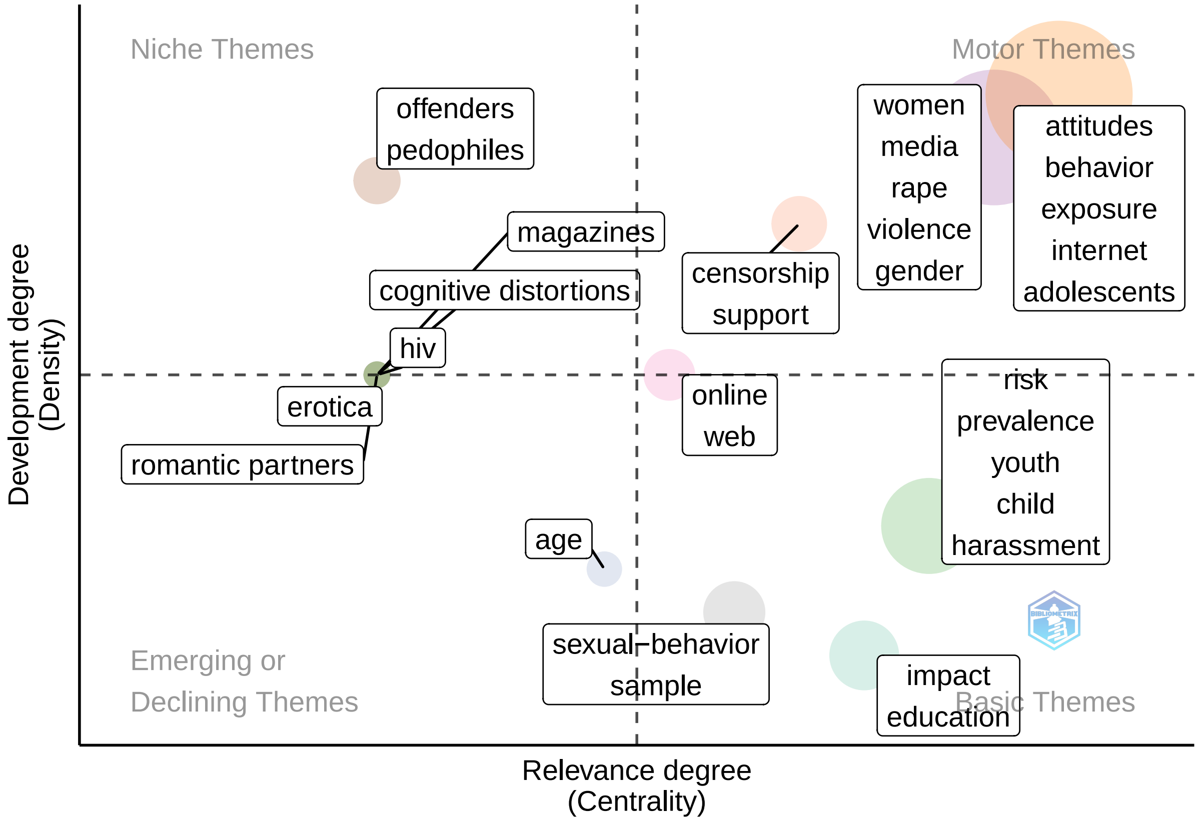

P2 (2011-2019)

Male

```
pmp2 <- male_p2 %>%
  thematicMap(field = "ID", n=300, minfreq = 10, stemming = TRUE,
              size = 0.3, n.labels = 5)

plot(pmp2$map)
```

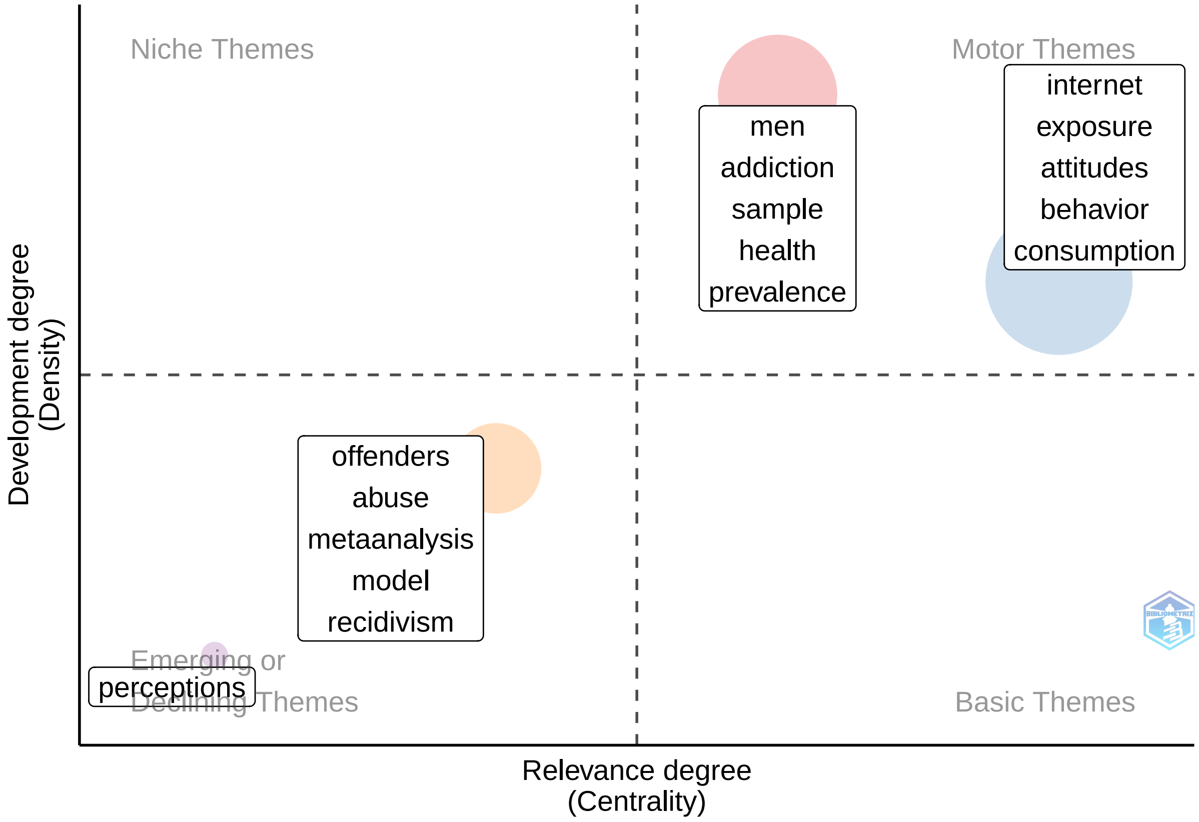

Female

```r
pfp2 <- female_p2 %>%
  thematicMap(field = "ID", n=300, minfreq = 10, stemming = TRUE,
              size = 0.3, n.labels = 5)

plot(pfp2$map)
```

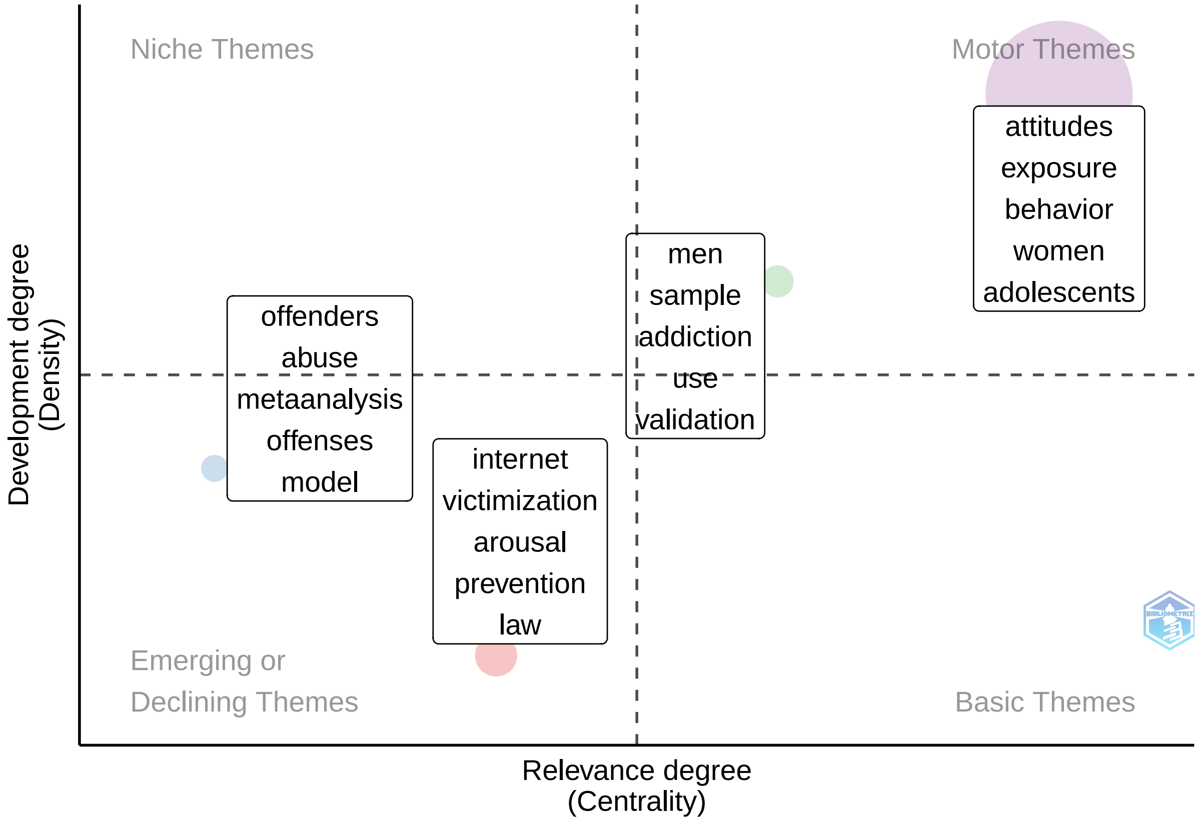

P3 (2020-2024)

Male

```
pmp3 <- male_p3 %>%
  thematicMap(field = "ID", n=300, minfreq = 10, stemming = TRUE,
              size = 0.3, n.labels = 5)
plot(pmp3$map)
```

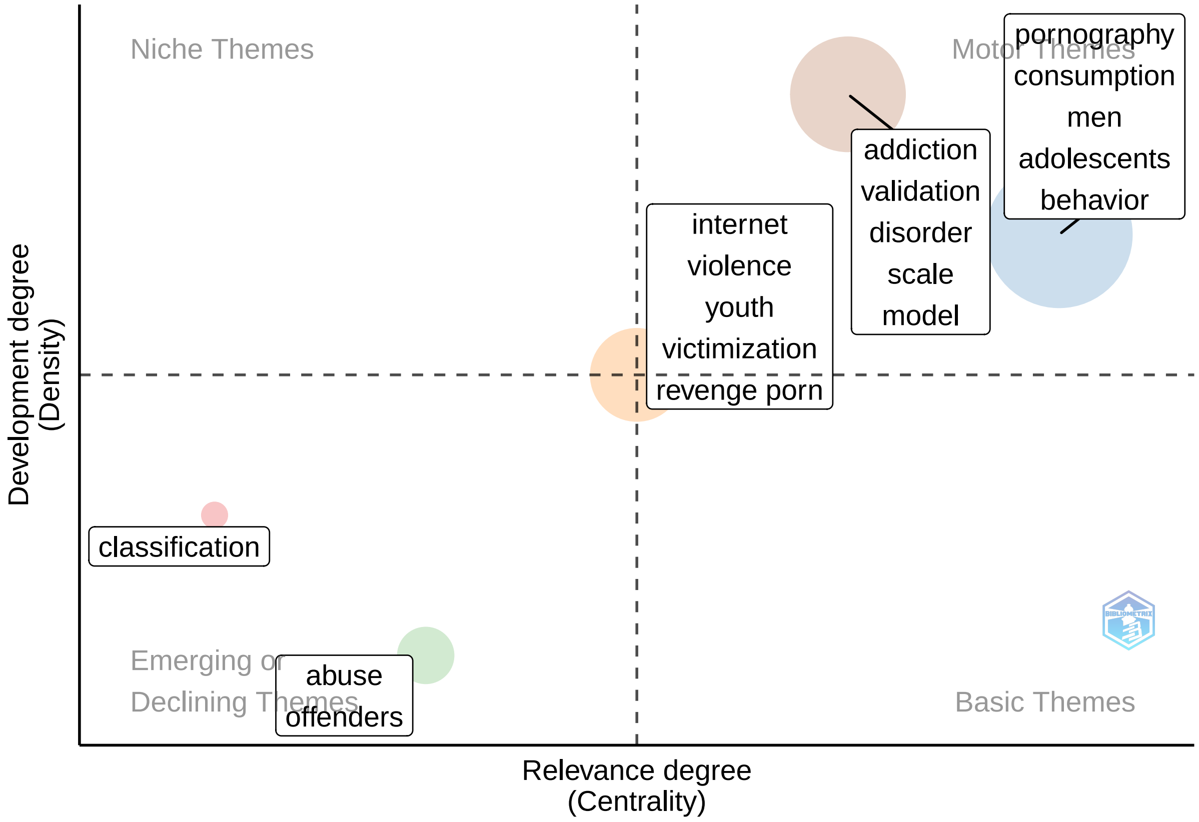

Female

```
pfp3 <- female_p3 %>%
  thematicMap(field = "ID", n=300, minfreq = 10, stemming = TRUE,
              size = 0.3, n.labels = 5)
plot(pfp3$map)
```

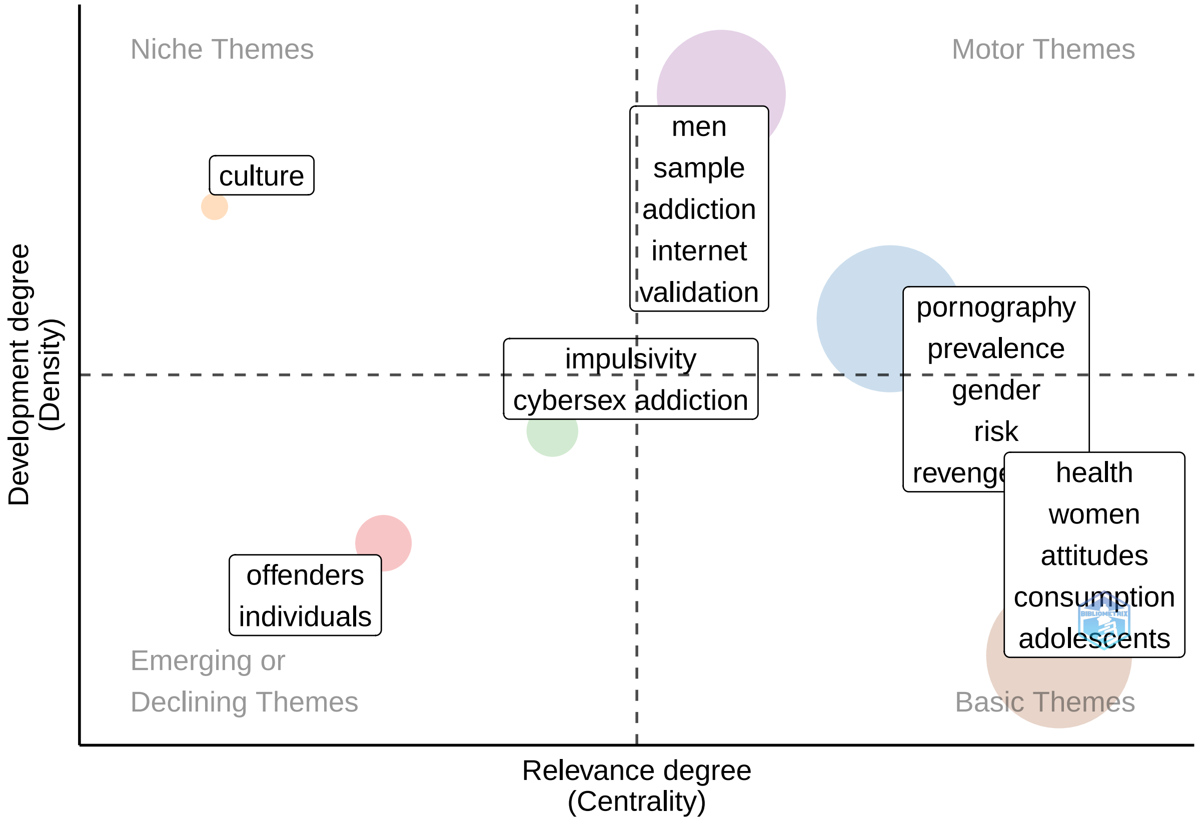

Niche Themes
Motor Themes
culture
men
sample
addiction
internet
validation
pornography
prevalence
gender
risk
revenge
impulsivity
cybersex addiction
health
women
attitudes
consumption
adolescents
offenders
individuals
Emerging or
Declining Themes
Basic Themes
Development degree
(Density)
Relevance degree
(Centrality)

Interpretation of the Overall Results (2001-2024)

This section describes the results of our overarching analysis that considers the entire time period (2001-2024). The remaining analysis can be found in the main text of the manuscript.

Overarching research topics are displayed in the Reproducibility Report above. Several topics were common for both male- and female-led publications; however, their relevance and cohesiveness varied. The shared themes within research topics concerned abuse and offenders that can be described as emerging or declining themes (i.e., peripheral and undeveloped), with higher relevance for women (offenders, offenses and abuse, meta-analysis, model) than for men (offenders, abuse). Another partially shared theme concerned exposure to and attitudes toward pornography and was classified as a motor theme (i.e., central and developed) in female-led articles (attitudes, women, pornography, exposure, behavior) and as a basic theme (i.e., central but undeveloped; exposure, attitudes, women, impact, media) in male-led articles.

Beyond these common research interests, several key differences emerged over the period covered by our sample. Namely, violence sometimes ascribed to pornographic content was a motor theme (i.e., central and developed; internet, violence, victimization, revenge porn, rape) in female-led publications – a topic that was largely absent in male-led research. In contrast, research topics on support and recidivism were clustered as niche themes (i.e., developed but peripheral) and appeared exclusively in male-led publications. Additionally, male-led research had several basic themes that were central but undeveloped (e.g., internet, behavior, pornography, risk, meta-analysis and consumption, men, adolescents, gender, sex), but only one motor theme (addiction, disorder, scale, validation, validity).
